# Zooming in on the molecular characteristics of swine influenza virus circulating in Colombia before and after the H1N1pdm09 virus

**DOI:** 10.3389/fvets.2022.983304

**Published:** 2022-09-21

**Authors:** William F. Osorio-Zambrano, Andres F. Ospina-Jimenez, Santiago Alvarez-Munoz, Arlen P. Gomez, Gloria C. Ramirez-Nieto

**Affiliations:** Microbiology and Epidemiology Research Group, Facultad de Medicina Veterinaria y de Zootecnia, Universidad Nacional de Colombia, Bogotá, Colombia

**Keywords:** RNA virus, South America, swine influenza, swine viruses, viral zoonosis

## Abstract

Influenza is one of the most critical viral agents involved in the respiratory disease complex affecting swine production systems worldwide. Despite the absence of vaccination against swine influenza virus in Colombia, the serologic reactivity to classic H1N1 and H3N2 subtypes reported since 1971 indicates the virus has been circulating in the country's swine population for several decades. However, successful isolation and sequencing of field virus from pigs was nonexistent until 2008, when H1N1 classical influenza virus was identified. One year later, due to the emergence of the influenza A (H1N1) pdm09 virus, responsible for the first global flu pandemic of the 21st century, it was introduced in the country. Therefore, to understand the impact of the introduction of the H1N1pdm09 virus in Colombia on the complexity and dynamics of influenza viruses previously present in the swine population, we carried out a study aiming to characterize circulating viruses genetically and establish possible reassortment events that might have happened between endemic influenza viruses before and after the introduction of the pandemic virus. A phylogenetic analysis of ten swine influenza virus isolates from porcine samples obtained between 2008 and 2015 was conducted. As a result, a displacement of the classical swine influenza virus with the pdmH1N1 virus in the swine population was confirmed. Once established, the pandemic subtype exhibited phylogenetic segregation based on a geographic pattern in all the evaluated segments. The evidence presents reassortment events with classic viruses in one of the first H1N1pdm09 isolates. Thus, this study demonstrates complex competition dynamics and variations in Colombian swine viruses through Drift and Shift.

## Introduction

Influenza virus is one of the major causes of respiratory disease in swine often characterized by a rapid onset of high fever, lethargy, loss of appetite, labored breathing, and coughing. Although mortality is low and most animals recover in approximately a week, weight loss can be severe. In addition, Swine Influenza Virus (SIV) can contribute to the occurrence of more chronic respiratory disease problems associated with other viruses and bacteria similar to humans. Due to its clinical and economic effects, influenza in pigs substantially impacts the swine meat industry globally (1). Given the zoonotic characteristics and pandemic potential of this virus, it represents a continuous threat to human health. This viral disease in pigs is naturally caused by a virus from the genera Influenza Virus A (IAV) or *Alphainfluenzavirus*, a member of the Orthomyxoviridae family (2). IAVs are classified into subtypes according to their combination of surface glycoproteins Hemagglutinin (HA) and Neuraminidase (NA), for which 16 subtypes of HA (H1-16) and nine of NA (N1-9) are globally recognized to be present in avian reservoirs. In addition, there are two HA subtypes (H17-18) and two NA subtypes (N10-11) present in American bats (3, 4). The characteristics of the negative ssRNA type genome of IAV have been associated with constant evolution through the accumulation of mutations (Drifts) and genetic reassorting effect (Shifts) (5–7).

Swine are susceptible to IAVs adapted to humans and birds, although the main subtypes that affect this species are H1N1, H1N2, and H3N2. This susceptibility happens due to the expression in the swine of complex carbohydrate receptors of sialic acid that bind to a galactose residue in an α2,3Gal and α2,6Gal linkage, combined with the properties of the proteins swANP32A and swANP32B located in the swine respiratory tract (8, 9). These characteristics allow pigs to act as intermediate hosts where adaptative and variation processes due to Drift or Shift occur (10, 11), increasing the chances of viral evolution of viruses of severe public health concern such as the H1N1 of 2009 (H1N1pdm09) (12–14).

Among the most predominant swine subtypes, a vast genetic and antigenic diversity is associated with interspecies jump events and geographic and temporal patterns (4, 15–17). A recent analysis of the H1 subtype demonstrated how the principal swine lineages spread to new geographic regions and evolved to different clades after their establishment in pigs, albeit influenced by geographic barriers and animal movement patterns; viruses from a North American origin have disseminated to all continents while viruses from European source are mainly distributed across Asia (16).

Before 2009, shared knowledge of IAV in swine was limited to North America and Eurasia, where dominance was related to H1N1 classic (1A), H1N1 Eurasian (1C), H1 Human-like (1B), and H3N2 Human-like lineages with a predominance of reassortant viruses carriers of Triple-Reassortant Internal Gene (TRIG) in North America and of internal genes from Euroasian lineage in Europe (18–22). After the introduction of the H1N1pdm09 to the global viral dynamic, its dominance and subsequent reassortment with endemic subtypes was observed. North America has shown a tendency to increase the number of variants carrying genes M and NP of H1N1pdm09 along with TRIG genes and combinations of HA and NA (23–25). Likewise, in Latin American countries such as Mexico, Brazil, and Argentina, viruses that carry internal genes of H1N1pdm09 have also been reported (26–30).

Despite the high importance of swine meat production in Colombia, the dynamics of IAV in that country are still mostly unknown. The swine population is primarily found in commercial production systems (76.9%) located in the Antioquia, Valle del Cauca, Cundinamarca, and Meta regions, where evidence of IAV circulation has been collected (31–34). Currently, vaccination against influenza in pigs in Colombia is non-existent; thus, serologic reactivity is indicative that subtypes H3N2 and classic H1N1 have been circulating in Antioquia since 1971 (35) and H3N2 in Valle del Cauca, Cundinamarca, Meta, and other regions since the early 2000s (36). The analyses of the first viral isolates in the country confirmed the presence of classic H1N1 from clade 1A in Antioquia in 2008 (34), as well as the introduction and dissemination of H1N1pdm09 to other major pig-producing regions since 2009 (33, 37). Subsequent investigations validate the constant circulation of IAV, specifically H1N1pdm09 in Antioquia, Cundinamarca, Valle del Cauca, Meta, and the “Coffee Region” (formed by Caldas, Huila, Quindío, and Risaralda regions) (32, 38, 39). However, the change in IAV viral dynamics in the Colombian swine population after the introduction of H1N1pdm09 remains unclear. To this date, there are no investigations where the characterization of internal genes has been assessed. Therefore, we carried out a study aiming to genetically characterize circulating viruses in pigs from 2008 to 2015 in Colombia and establish the possible events that occurred in endemic influenza viruses after the introduction of pandemic virus. This paper presents results that contribute to understand the impact of the introduction of H1N1pdm09 virus in Colombian swine herds and the dynamics of influenza viruses in the pig population.

## Materials and methods

### Sample collection and virus isolation

For this study, 10 isolates were selected from the viral repository of the Laboratory of Virology of the Universidad Nacional de Colombia, obtained between 2008 and 2015 from commercial pigs located in regions with a high production of swine meat in Colombia (Table 1). Such isolates come from nasal swabs [2008–2010] collected from live animals, or pulmonary tissue samples that were taken at pig slaughterhouses [2015] (37, 38). Viral isolation was performed using MDCK cells or 10-day-old SPF embryonated chicken eggs. Cell culture supernatant or allantoid fluids were harvested after an incubation period of 72 hours at 37°C; then, IAV presence was confirmed in a hemagglutination test using 0.5% chicken red blood cells and RT-PCR analysis for detection of influenza A matrix gene. Three blind passages were used for isolation trials of each sample. Samples were collected, causing minimum distress on analyzed pigs and following the mandatory biosecurity measures. Procedures and conditions used to obtain samples were approved by the bioethics committee of the school of Veterinary Medicine and Animal Science of the National University of Colombia.

**Table 1:** Geographical origin and type of sample of the Influenza A Virus isolates (H1N1) from the Virology Laboratory collection included in this study.

**Strain Name**	**Isolation region**	**Isolation source**	**Global Swine H1 Clade**
A/swine/Colombia/0401/2008	Antioquia	Nasal swab	1A.1
A/swine/Colombia/0801/2008	Antioquia	Nasal swab	1A.1
A/swine/Colombia/1-01/2009	Valle del Cauca	Nasal swab	1A.3.3.2
A/swine/Antioquia/0201/2009	Antioquia	Nasal swab	1A.3.3.2
A/swine/Colombia/1503/2010	Cundinamarca	Nasal swab	1A.3.3.2
A/swine/ Antioquia/3-015/2015	Antioquia	Lung	1A.3.3.2
A/swine/Cundinamarca/1-019/2015	Cundinamarca	Lung	1A.3.3.2
A/swine/Antioquia /3-020/2015	Antioquia	Lung	1A.3.3.2
A/swine/Antioquia /3-021/2015	Antioquia	Lung	1A.3.3.2
A/swine/Antioquia /3-027/2015	Antioquia	Lung	1A.3.3.2

### RNA extraction and characterization using RT-PCR

Total RNA extraction was obtained from an aliquot of each viral isolate sample (2008–2010) using a RNeasy Mini Kit (Qiagen^®^) according to the manufacturer's instructions. For the isolates obtained in 2015, we used a High Pure Viral Nucleic Acid Kit (Roche Life Science^®^). cDNA synthesis from viral RNA was achieved through RT-PCR using Uni12 (Invitrogen^®^), 5'—GGGGGGAGCAAAAGCAGG-3' (40), and employing SuperScript™ III Reverse Transcriptase enzyme (Invitrogen^®^).

One-step RT-qPCR reactions were performed on LightCycler 480 real-time thermocycler (Roche Life Science^®^) utilizing the SuperScript™ III Platinum One-step Quantitative RT-PCR System (Invitrogen^®^). RT-qPCR was conducted using TaqMan hydrolysis probes for detection of influenza A virus (Inf A) M gene, swine influenza (SW Inf A) virus NP gene, and H1N1 swine (SW H1) influenza HA gene, following the Centers for Disease Control and Prevention (CDC) protocols (Table S1) (41). Further subtyping was performed by multiplex RT-qPCR analysis using TaqMan hydrolysis probe to detect HA and NA genes of swine H3N2 viruses, adapting the Standard Operating Protocol provided by the University of Minnesota—USA (MOL.SOP.218, 2008). Briefly, 25 μL master mix reactions with 1X of Reaction Mix Invitrogen^®^, 0.5 μM of H3 forward primer, 0.75 μM of H3 Reverse, 0.75 μM of N2 forward, 1 μM of N2 Reverse, 0.3 μM of each probe (Table S1), 0.5 μL of Superscript III RT/Platinum^®^ Taq Mix, and 5 μL of RNA were used. Reactions were carried out at 50°C for 30 min, 95°C for 15 min, and 35 cycles of 94°C for 30 s, 58°C for 1 min, 72°C for 1 min, and a final elongation step of 72°C for 7 min. Data analysis and recovery were carried out in Roche LightCycler software version 1.5. Results were considered negative when samples exhibited *Cp*-values >38 and positive when the value was below the mentioned limit.

### Sequencing and phylogenetic analysis

The approach used to amplify the analyzed genes consisted of dividing the gene into two fragments and using the universal oligonucleotides reported by Hoffman et al. (40) with internal primers donated by the Influenza Research Laboratory of the University of Maryland—USA (Table S1).

The PCR products from the amplification of HA, NA, M, and NS genes were revealed in 1% agarose gel, purified using Qiagen^®^ kit for gel extraction (Valencia, CA) based on manufacturer's instructions, and sequenced through the Sanger method in Macrogen USA^®^ online sequencing order system using Big- Dye^®^ Terminator Cycle Sequencing. DNA sequences were combined and edited using the Lasergen sequence analysis package (DNAStar V7.1, Madison, WI, USA). Data regarding these isolates and sequence access numbers of the analyzed segments are shown in Table S2.

For the phylogenetic analyses, these sequences were aligned with previously reported sequences from around the world and from different time points, including some obtained from humans and pigs of H1N1 and H3N2 subtypes, using the MUSCLE program (Multiple Sequence Comparison by Log-Expectation), accessible in the Influenza Research Database (IRD) (42) in conjunction with the Uclust program.

The visualization and exploration of the annotated phylogenetic trees in the IRD platform were possible using the Archaeopteryx.js software tool. Phylogenetic trees were constructed with RAXML and calculated based on 1,000 bootstrap replicates, also available in IRD (42). In the analysis, IAV reference sequences from the National Center for Biotechnology Information Database (NCBI) were included (Table S3). Besides the classic H1N1 viral isolate identified as A/swine/Colombia/0401/2008, which has been reported previously, a second isolate from 2008 was included (A/swine/Colombia/0801/2008), even though it has not been reported until now (FASTA sequence is shown in Table S4).

## Results

### Phylogenetic analysis of the HA segment

**Figure 1:**
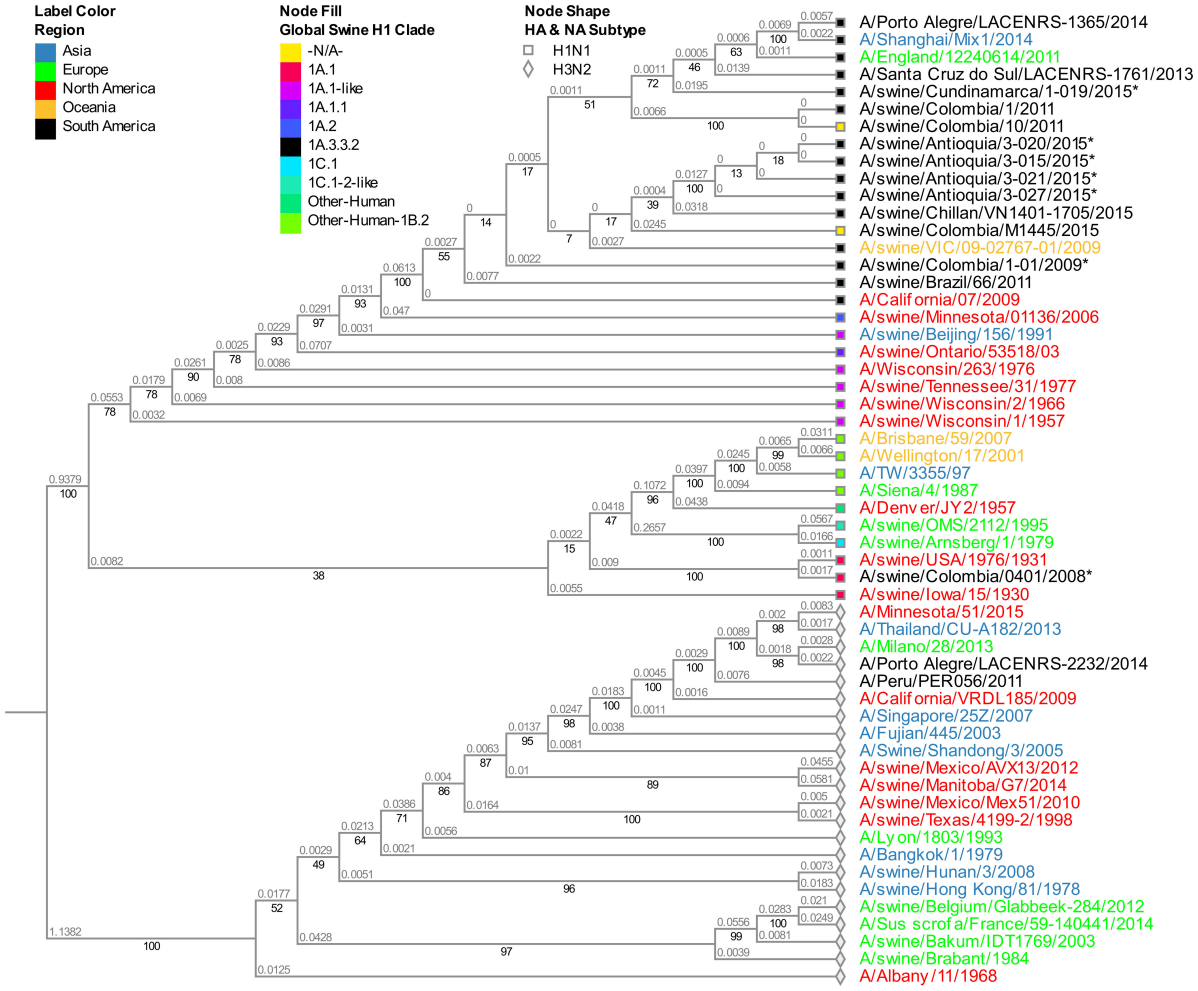
Phylogenetic analysis for HA gene of swine influenza A virus. Phylogenetic tree was generated using RAxML program for maximum likelihood-based inference. The reliability of the trees was inferred by bootstrap analysis with 1,000 replications. The tree was constructed with representative sequences from human and swine of H1N1 classical and H1N1pdm09 and human and swine H3N2. Colombian isolates included on this study are labeled with asterisks.

The phylogenetic analysis indicates that the 10 viral isolates included in the study are classified as the H1 subtype. There is an evident separation between the isolates obtained in 2008, all grouped as part of the classic swine lineage (1A.1), and the ones obtained after 2009, which correspond to viruses from the H1N1pdm009 pandemic clade 1A.3.3.2 (Figure 1 and Figure S1).

Both viral isolates from 2008 (A/swine/Colombia/0401/2008 and A/swine/Colombia/0801/2008) are similar and are allocated in the North American clade 1A.1, presenting high similarity with old swine viruses from North America from the decade of the 1930s (A/swine/USA/1979/1931 and A/swine/Iowa/15/1930) (Figure 1 and Figure S1). All the isolates collected after 2009 are in the global swine clade 1A.3.3.2. From these, 2009 viruses have higher phylogenetic proximity to the reference virus A/Cal/07/2009, while the isolates from 2010 and 2015 are less related to it and are grouped differently in the phylogenetic tree according to their origin. The viruses from the Antioquia region of 2015 and one from Cundinamarca of 2010 are organized next to swine viruses from Chile and Australia. On the other hand, Cundinamarca 2015 isolate is closely related to human viruses from Brazil, Europe, and Asia (Figure 1 and Figure S1).

These results show the presence of H1 virus from classic swine lineage 1A in pigs in Colombia in 2008 and suggest that since the introduction of the H1N1pdm2009 virus to the country, the dominance of the pandemic virus remained constant in the evaluated regions at least until 2015 presenting some level of variation over time.

### Phylogenetic analysis of the NA segment

**Figure 2:**
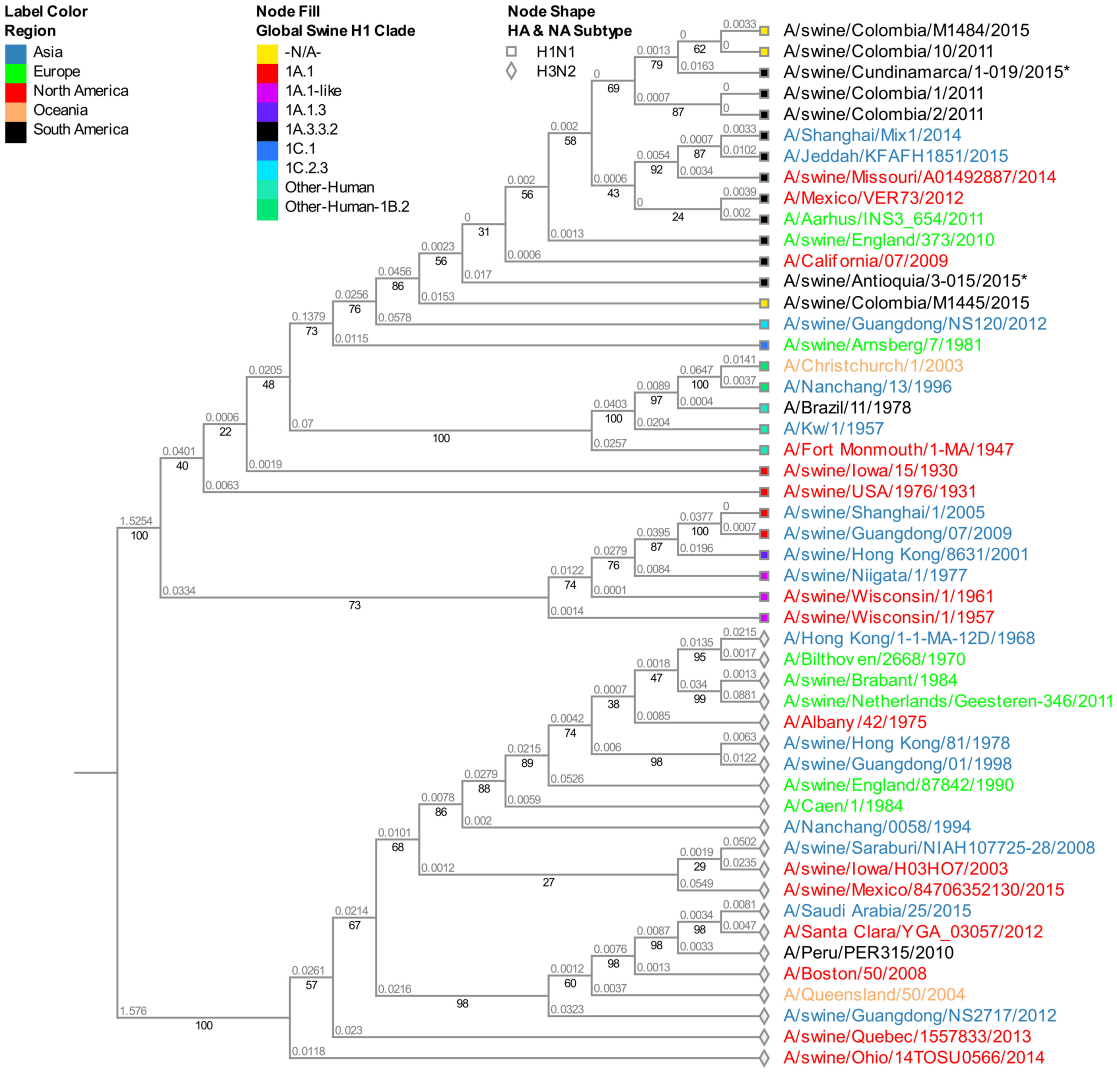
Phylogenetic analysis for NA gene of swine influenza A virus. Phylogenetic tree was generated using RAxML program for maximum likelihood-based inference. The reliability of the trees was inferred by bootstrap analysis with 1,000 replications. The tree was constructed with representative sequences from human and swine H1N1 classical and H1N1pdm09, as well as human and swine H3N2. Colombian isolates included on this study are labeled with asterisks.

The viruses evaluated in the study correspond to the N1 subtype. Virus A/swine/Colombia/0801/2008 presents high similitude again with the old North American virus A/swine/USA/1976/1931 (Figure S2). Given that the four sequences from 2015 obtained in the Antioquia region (3–15, 3–20, 3–21, and 3–27) have identical sequences for this segment, for the phylogenetic tree construction only isolate A/swine/Antioquia/3-015/2015 was included. Regarding the viruses collected in 2015, the same separative tendency based on geographical origin observed with the HA segment remains. The isolates from Antioquia are more closely related to the virus of reference A/Cal/07/2009 than the one obtained in Cundinamarca in 2015 (A/swine/Cundinamarca/1-019/2015). This pandemic H1N1 Cundinamarca 2015 virus is grouped separately, and contrary to what was noticed with the HA segment, there is a greater relatedness to NA from swine viruses previously reported in Colombia (Figure 2).

### Phylogenetic analysis of the M segment

**Figure 3:**
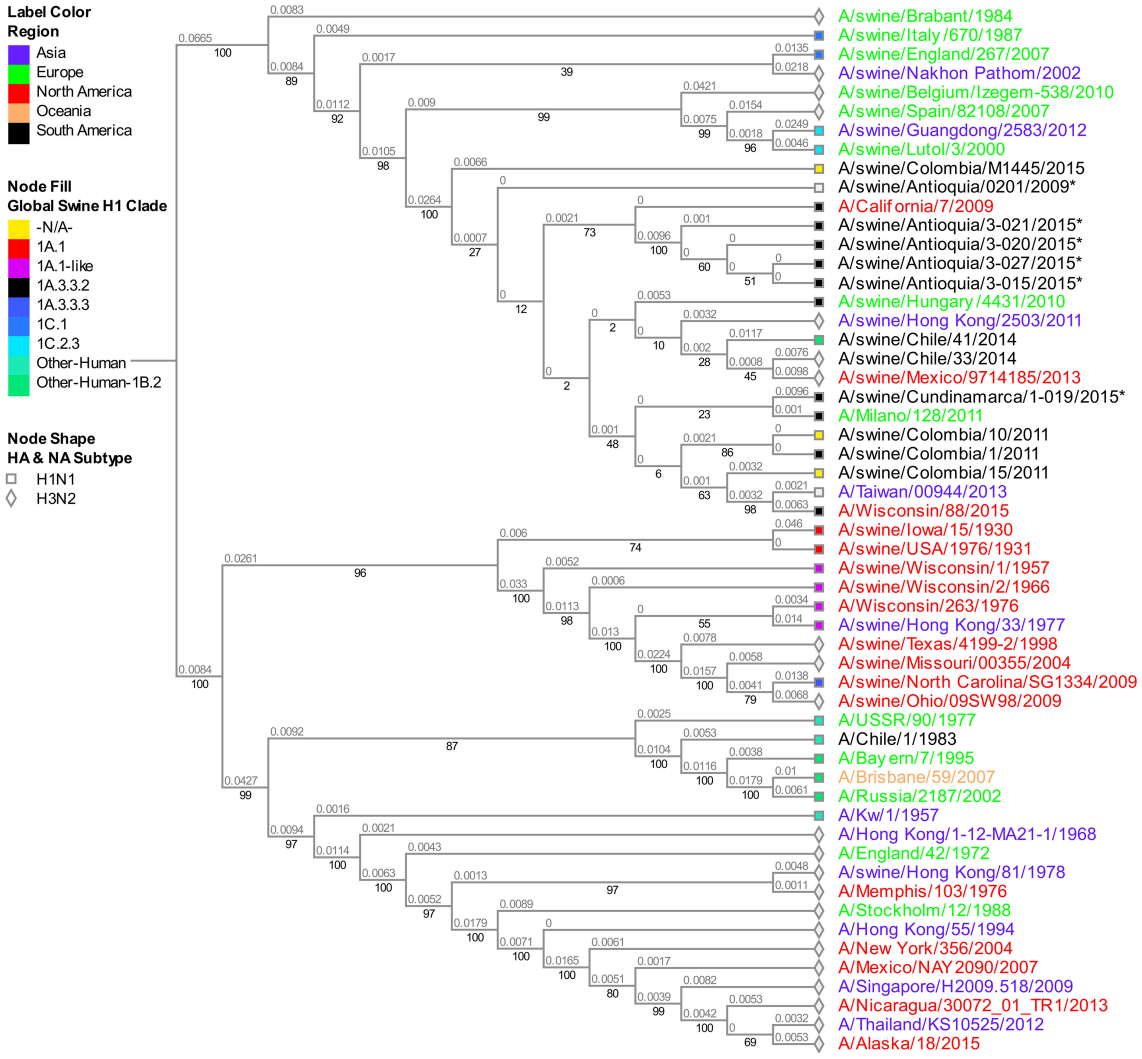
Phylogenetic analysis for M gene of swine influenza A virus. Phylogenetic tree was generated using RAxML program for maximum likelihood-based inference. The reliability of the trees was inferred by bootstrap analysis with 1,000 replications. The tree was constructed with representative sequences from human and swine H1N1 classical and H1N1pdm09, as well as human and swine H3N2. Colombian isolates included on this study are labeled with asterisks.

Albeit being one of the most stable segments in IAV, we still observed the differentiation pattern by region of origin. The isolate from Cundinamarca 2015 is distinctively grouped and closely related to a human pandemic isolate from 2011 (A/Milano/128/2011). On the other hand, all four viruses obtained in 2015 from the Antioquia region are grouped in the same subclade showing high proximity with the human pandemic reference virus of 2009 A/Cal/07/2009. Among the Antioquia 2015 viruses, noticeably, there is a slight difference with A/swine/Antioquia/3-015/2015, which phylogenetically separates it from the other three isolates (Figure 3).

Furthermore, the 2008 classic virus A/swine/Colombia/0401/2008 obtained in Antioquia is identical to the pandemic virus collected the following year in the same region (A/swine/Antioquia/0201/2009). Even though the samples are phylogenetically separated from the A/Cal/07/2009 virus, they are all within the same cluster of Eurasian-type M sequences (Figure S3).

### Phylogenetic analysis of the NS segment

**Figure 4:**
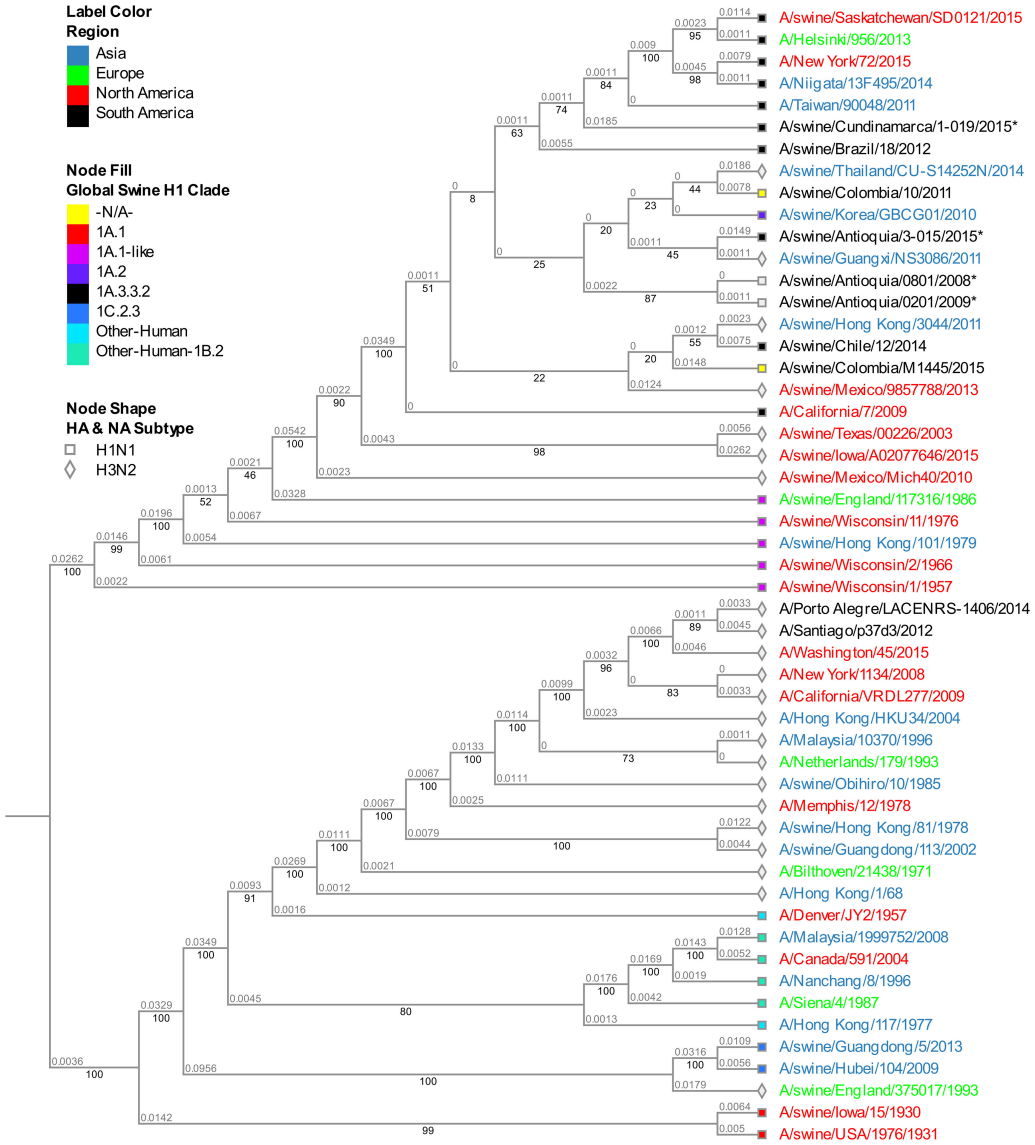
Phylogenetic analysis for NS gene of swine influenza A virus. Phylogenetic tree was generated using RAxML program for maximum likelihood-based inference. The reliability of the trees was inferred by bootstrap analysis with 1,000 replications. The tree was constructed with representative sequences from human and swine H1N1 classical and H1N1pdm09, as well as human and swine H3N2. Colombian isolates included on this study are labeled with asterisks.

The analysis of this segment demonstrates the relationship of the viruses collected in 2015 with the virus of reference A/Cal/07/2009, keeping the separative tendency based on the region of origin (Figure 4). The virus from Cundinamarca 2015 is persistently associated with human viruses identified in Asia, North America, Northern Europe, and one Brazilian swine virus, unlike the 2015 isolates from Antioquia which are grouped only with swine viruses. Additionally, the NS segment of the classic 2008 isolate (A/swine/Antioquia/0801/2008) shows high phylogenetic proximity to the pandemic sample taken in the same region 1 year latter (A/Swine/Antioquia/0201/2009), grouping them in one subclade.

## Discussion

Using the results provided in this study, we demonstrated that the 2008 Colombian isolates carry an HA segment from the North American clade 1.A.1 classic lineage and are similar to old North American viruses gathered in 1930–1931, suggesting stability over time until the introduction of a new virus responsible for the 2009 pandemic. Moreover, it is clear that after 2009 all the studied isolates had external protein genes from pandemic clade 1A.3.3.2, an expected outcome taking into account that the Colombian swine population was susceptible to the infection of this new virus, same as what happened across the globe. It is also worth mentioning the relevance of the results obtained from the viruses isolated in 2015, where phylogenetic segregation in the evaluated segments related to the region of origin is evident, implying that patterns of adaptation and new dynamics could be associated with geographical or environmental factors resulting in viral selection, a product of the balance of the host-virus interaction. The latter is visible when analyzing the behavior of the Antioquia 2015 viruses having high similarity among them and a strong relationship with swine viruses reported worldwide; contrary to the observation described with Cundinamarca 2015 virus in which the segments HA, M, and NS present a more significant relationship to human viruses. This finding renders questions about the involvement of the human-animal interphase, a concept that requires further analysis. However, due to the limitations of this study and the minimal availability of information regarding the molecular characteristics of influenza virus circulating in the human population in the country, it is unattainable to determine. Thus, this lack of data begs the need for more studies of this virus in Colombia and ideally with the approach of ONE HEALTH (43).

Another captivating finding that requires further studies is the relationship between the classic viruses from 2008 and the pandemic virus (A/swine/Antioquia/0201/2009) at the M and NS genes, which suggests the possibility of events in the country before the introduction of the 2009 pandemic virus, elucidating potential mechanisms for the generation of viruses with new characteristics in the Colombian swine population or the conception of gene reassortment as seen after the introduction of the H1N1pdm09 virus. Both possible mechanisms reassert the importance of constantly tracking the molecular characteristics circulating in pig populations worldwide to anticipate new epidemics/pandemics generated by the influenza virus.

In contrast to the external segments, there is no phylogenetic separation between H1N1 and H3N2 subtypes in the M segment. This indicates that the phylogenetic evolutionary dynamics are different and independent of the ones observed in HA and NA. When discovering phylogenetic similarity between the different subtypes and clades (Global swine H1 clade), an argument could be made about segment M tending to conserve itself more than HA and NA, probably due to the high level of stability of the proteins it generates, necessary for viral survival and prevalence, a concept that seems non-definitive for external segments which present high change rate (44).

It is important to highlight that the main objective of this study is to provide the only existing information on the characteristics of circulating viruses before, during, and after the entry of the H1N1pdm09 virus. At this point this information is especially relevant because the included viruses are the only existing isolates that were taken precisely before and immediately after the emergence of the H1N1pdm09 virus in the world. The isolates were only obtained from some regions, a situation that limited our analyzes to isolates obtained, however these regions concentrate the highest proportion of the pig population in Colombia.

**Figure 5:**
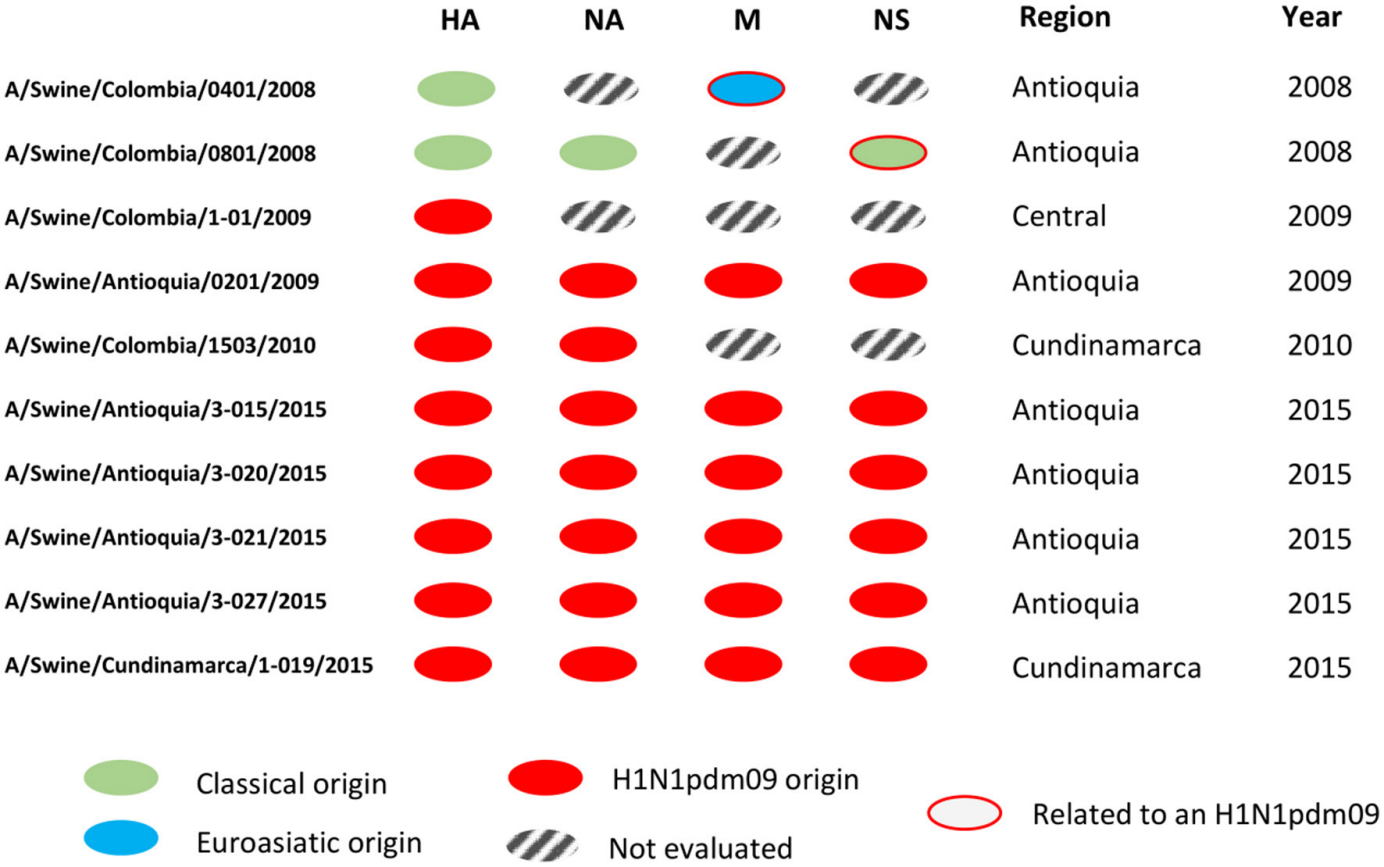
Genomic segment constellations of Colombian swine influenza A viruses analyzed in this study. Genetic configurations of internal genes from H1N1pdm09 origin in the viruses identified as the H1N1 subtype.

In this sense, this study contributes to the general knowledge of the swine influenza virus, filling a gap regarding the molecular characteristics of the virus circulating in the past in a non-existent vaccination country where the major evidence came from serologic activity as observed in previous studies (35, 36). Even though we understand the number of isolates analyzed in the study, particularly before 2009, as a limitation, it is the availability of these isolates (valid per IRD criteria), including the ones obtained in 2009, that confer additional value since they represent the only existent information allowing us to understand the situation before, during, and after the entry of H1N1pdm09 virus in the country, information which is scarcely available around the world. Figure 5 aids in visualizing the genetic configurations of internal genes predominantly from H1N1pdm09 origin in the viruses identified as the H1N1 subtype.

We hypothesize that the stability of the classic lineage for at least 37 years (35) was due to the low pressure on immunologic selection by the non-existent vaccination and the inherent dynamics of swine populations in production systems (45, 46), developing a persistence with no significant variations in the viral genome. This has been observed in regions like North America, where the lineage remains steady for around 70 years (47–49). Such characteristic has been associated with the ancientness of the virus in the swine host, by which old subtypes present fewer variations than recent ones. This phenomenon leads to a higher pressure of evolutionary adaptation that could have influenced the stability of the virus in Colombia (50). Segment NA of virus A/Swine/Colombia/0801/2008 shows the same stability (Figure S2).

With the internal segments evaluated in the study, we determined the relationship of these viruses with pandemic viruses detected afterward (Figure S3, Figures 4, 5). In the gene M of A/Swine/Colombia/0401/2008 exists similarity with the segment of A/Swine/Colombia/0201/2009 (Figure 5), both situated in the corresponding M gene cluster of pandemic and Euroasian viruses (Figure S3). Such discovery proposes the presence of Eurasian genes in classic Colombian viruses before the introduction of the H1N1pdm09 virus to the country, a finding that has not been reported before since circulation of the virus has only been registered in Mexico within the American continent (28, 51). It is plausible that Euroasian segments have been circulating in swine viruses previously in Colombia and other Latin American countries undetected; this is supported by the “unsampled pig herd” theory on the origin of H1N1pdm09 (52). Regarding the NS segment, similarity exists between viruses A/swine/Colombia/0801/2008 and A/swine/Antioquia/0201/2009 (Figure 4), possibly due to reassortment events that led H1N1pdm09 virus to acquire NS genes from classic Colombian viruses, having the same phylogenetic origin (53, 54). However, the segment was not detected in later pandemic isolates.

H1N1pdm09 viruses were introduced in the Colombian territory in 2009 (34), and since then, a clear dominance has been obeyed by being the only subtype detected. This indicates the displacement of the classic subtype due to a competitive dynamic (16, 55) in which H1N1pdm09 virus was more efficient in terms of higher viral fitness and antigenic difference after its recent installment (17, 56, 57). Such sustained superiority is contrary to what has been seen in other countries, where H1N1pdm09 circulates but not as the dominant subtype (15, 58, 59). In Colombia, this difference could be influenced by factors such as the non-existent vaccination or the apparent lower diversity in swine IAV characterized until now, although due to the lack of studies made in the country, there is not enough evidence to support this hypothesis.

Once H1N1pdm09 was established in the country, evolutionary processes, and variations by Drift in all genes and by Shift in internal genes occurred. These processes are most evident in the 2015 viruses, in which consistent phylogenetic grouping following geographic patterns is observed (Figures 1–4, Figures S1–S3) and noticed as well in the relationship of M and NS genes from virus A/swine/Antioquia/0201/2009 with classic viruses encountered in the same region (Figure S3, Figures 4, 5). Multiple studies with the H1N1 subtype reported variations among regions, where evidence of separate evolution could eventually generate new clades and subclades (16, 60).

The viruses obtained from the Antioquia region in 2015 present a substantial similarity in all their segments while separated from the Cundinamarca (A/swine/Cundinamarca/1-019/2015) isolate in which a tendency to group HA, M, and NS with human viruses is ascertained (Figures 1, 3, 4). This points out the possibility of viral introduction in swine due to reverse zoonosis, previously suggested by Nelson et al. (61) in Colombia, a situation reported with notable frequency (62, 63); however, this requires further investigation. This separative tendency is not witnessed in the NA segment considering the high proximity to Colombian swine viruses previously reported (Figure 2), indicating the virus could acquire NA from other H1N1pdm09 due to reassortment with homologous viruses (53).

Considering the low amount of data regarding the molecular characterization of IAV in pigs in Colombia, this investigation provides valuable information about the dynamics and situations before, during, and after the introduction of the H1N1pdm09 virus, within a context of no immunological selection pressure caused by vaccination. Although the number of samples evaluated was small and limited to specific regions, these are representative as they were obtained in different years in the most swine-meat-productive regions of the country (31) and correspond to the only isolates available to date. To our knowledge, this is the first study in which the characterization of internal genes of Colombian influenza viruses is conceived.

The results gathered in this study evidence the variation of the H1N1pdm09 virus by Drift in different genomic regions, indicating its possible reassortment with classic viruses and the circulation of Eurasian genomic segments during 2008 in Colombia. Still, for a better interpretation of these results, further investigations involving a larger number of samples from other regions may lead to viral identification and molecular characterization in the country. It is also essential to determine the status of classic viruses, which, even though they seem to have been displaced by the H1N1pdm09 virus, are probably still circulating in other regions along with the H3N2 virus from which only serological evidence is reported (35, 36). Correspondingly, further investigations should be carried out on different H1 and H3 human-like viruses in Colombia that have been demonstrated to be a consequence of the human introduction in countries like Mexico, Chile, and Brazil (30, 64–66), allowing for the assessment of the status of human and swine influenza helping to elucidate the impact of this virus to public and animal health. Additional studies are encouraged to help construct the possible timeline IAV has undergone in Latin America to further understand viral dynamics aimed at better preparedness of preventive epidemiological models and surveillance systems.

## Data availability statement

The datasets presented in this study can be found in online repositories. The names of the repository/repositories and accession number(s) can be found in the article/Supplementary material.

## Ethics statement

The animal study was reviewed and approved by procedures and conditions used to obtain samples were approved by the Bioethics Committee of the School of Veterinary Medicine and Animal Science of the National University of Colombia, according to the CB-039-2014 and CB-058-2016.

## Author contributions

GR-N: conceptualization, supervision, and project administration. WO-Z and GR-N: methodology. WO-Z: software. WO-Z, GR-N, AO-J, SA-M, and AG: formal analysis, data curation, and writing—original draft preparation. WO-Z, GR-N, AO-J, SA-M, and AG: writing—review and editing. All authors contributed to the article and approved the submitted version.

## Funding

Samples used in this study were obtained from projects funded by IICA-MADR-ACP-UNAL - Estudio Sobre la Influenza Porcina en Colombia: aislamiento, diagnóstico y control (Code number 202010010947) and the Departamento Administrativo de Ciencia, Tecnologia e Innovación (COLCIENCIAS 572-2014 code number 110165843183) in collaboration with the National Pork Board (Asociación Colombiana de Porcicultores-Fondo Nacional de Porcicultura) and the support of Universidad Nacional de Colombia.

## Conflict of interest

The authors declare that the research was conducted in the absence of any commercial or financial relationships that could be construed as a potential conflict of interest.

## Publisher's note

All claims expressed in this article are solely those of the authors and do not necessarily represent those of their affiliated organizations, or those of the publisher, the editors and the reviewers. Any product that may be evaluated in this article, or claim that may be made by its manufacturer, is not guaranteed or endorsed by the publisher.
