# Supplementary material for: Zooming in on the molecular characteristics of swine influenza virus circulating in Colombia before and after the H1N1pdm09 virus
Source: Front Vet Sci. 2022 Sep 21;9:983304. doi: 10.3389/fvets.2022.983304 (PMC9533064; doi:10.3389/fvets.2022.983304)
Supplement: Supplementary file 1 [file Data_Sheet_1.docx]

Supplementary Material

# Supplementary Figures and Tables

## Supplementary Figures


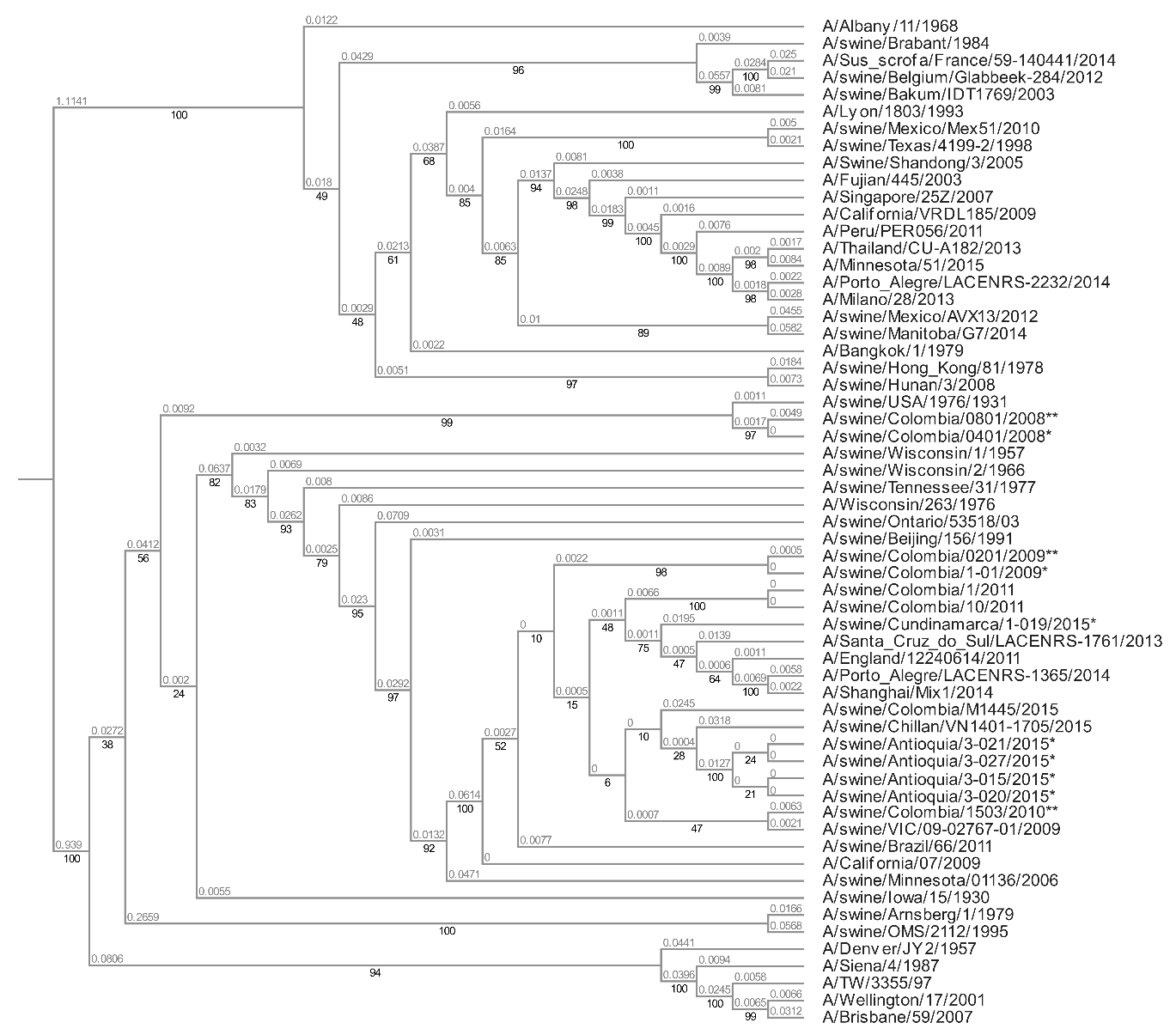


**Supplementary Figure 1.** Phylogenetic analysis for HA gene. Phylogenetic tree of HA gene generated using RAxML program for maximum likelihood-based inference. The reliability of the trees was inferred by bootstrap analysis with 1.000 replications. The tree was constructed with representative sequences from human and swine H1N1 classical and H1N1pdm09, and human and swine H3N2. Isolates from this study that were reported are labeled with one asterisk, and those that were not reported are labeled with two asterisks.


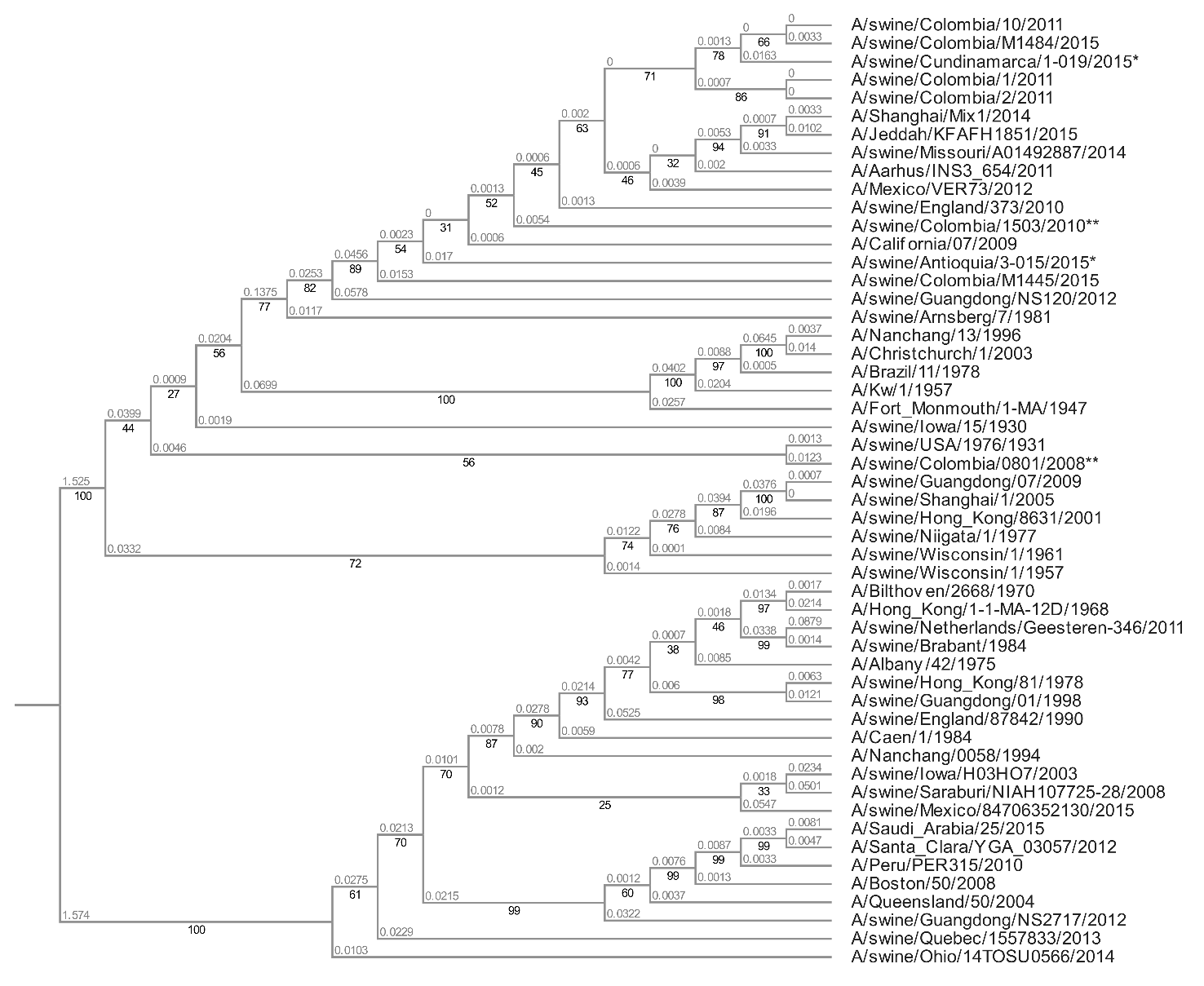


**Supplementary Figure 2.** Phylogenetic analysis for NA gene. Phylogenetic tree of NA gene generated using RAxML program for maximum likelihood-based inference. The reliability of the trees was inferred by bootstrap analysis with 1.000 replications. The tree was constructed with representative sequences from human and swine H1N1 classical and H1N1pdm09, and human and swine H3N2. Isolates from this study that were reported are labeled with one asterisk, and those that were not reported are labeled with two asterisks.


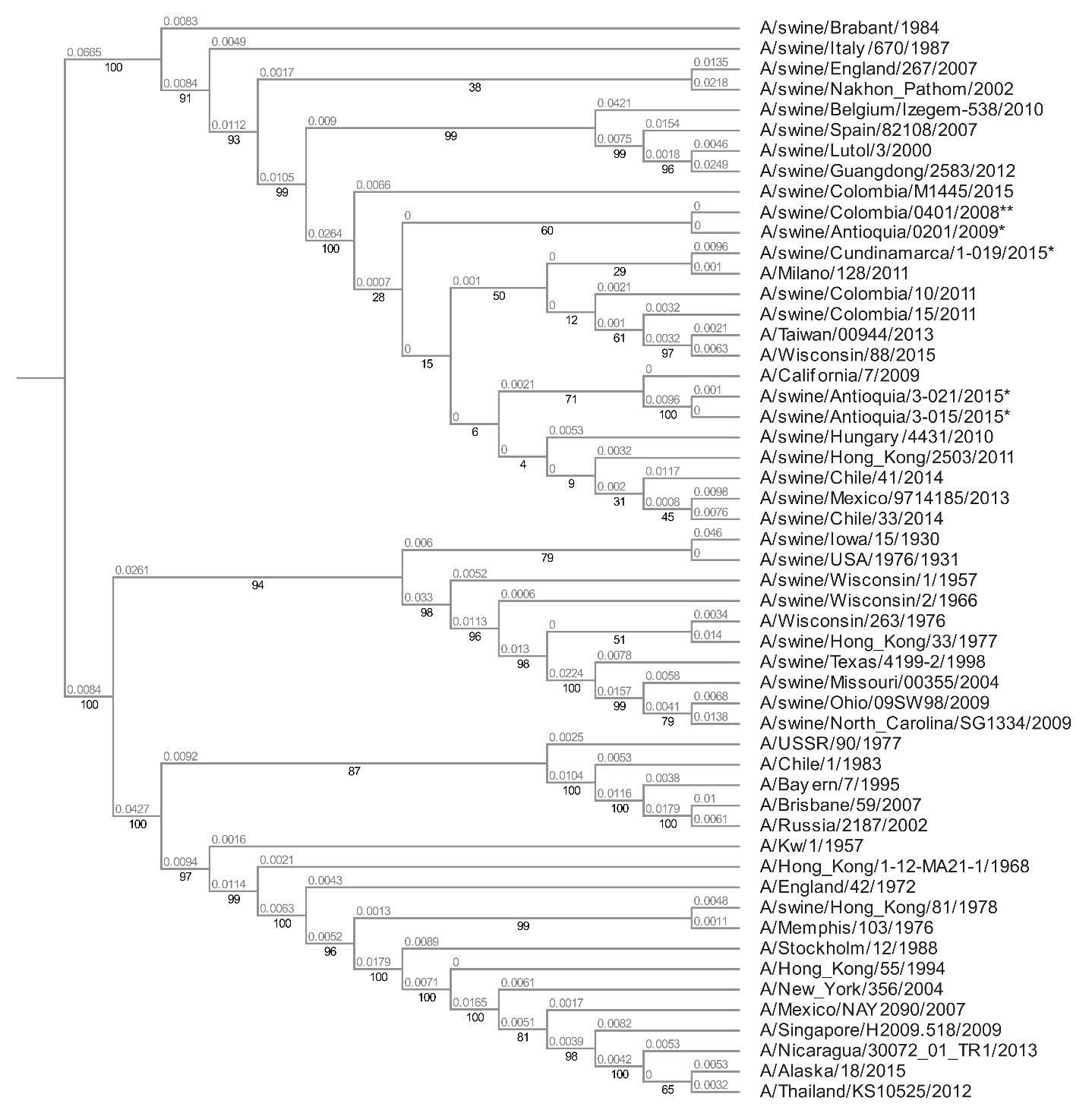


**Supplementary Figure 3.** Phylogenetic analysis for M gene. Phylogenetic tree of M gene generated using RAxML program for maximum likelihood-based inference. The reliability of the trees was inferred by bootstrap analysis with 1.000 replications. The tree was constructed with representative sequences from human and swine H1N1 classical and H1N1pdm09, and human and swine H3N2. Isolates from this study that were reported are labeled with one asterisk, and those that were not reported are labeled with two asterisks.

## Supplementary Tables

**Table S1.** Primers used for the amplification of swine influenza viruses.

| **Primer** | **Sequence 5’-3’** | **Target gene** | **Size (pb)** |
| --- | --- | --- | --- |
| **qRT-PCR primers according to CDC protocols^1^** | | | |
| Inf A Forward | GACCRATCCTGTCACCTCTGAC | M | 195 |
| Inf A Reverse | AGGGCATTYTGGACAAAKCGTCTA |  |  |
| Inf A Probe | TGCAGTCCTCGCTCACTGGGCACG |  |  |
| SW Inf A Reverse | GCACGGTCAGCACTTATYCTRAG | NP | 195 |
| SW Inf A Reverse | GTGRGCTGGGTTTTCATTTGGTC |  |  |
| SW Inf A Probe | CYACTGCAAGCCCATACACACAAGCAGGCA |  |  |
| SW H1 Forward | GTGCTATAAACACCAGCCTYCCA | H1pmd09 | 116 |
| SW H1 Reverse | CGGGATATTCCTTAATCCTGTRGG |  |  |
| SW H1 Probe | CAGAATATACATCCRGTCACAATTGGARAA |  |  |
| **qRT-PCR primers according to University of Minnesota protocol (MOL.SOP.218, 2008)** | | | |
| H3 Forward | CGCAATMGCAGGTTTCATAGA | H3 | 68 |
| H3 Reverse | GATGCCTGAAACCGTACCA |  |  |
| H3 Probe | ATGGTTGGGAGGGAAT |  |  |
| N2 Forward | TGGTCAAAGCCGCAATG | N2 | 52 |
| N2 Reverse | CAGCGGAAAGCCKAATCG |  |  |
| N2 Probe | ATTACAGGATTTGCACTTTTT |  |  |
| **PCR primers used for sequencing** | | | |
| BmHA1F^2^ | TATTCGTCTCAGGGAGCAAAAGCAGGGG | HA segment 1 | 931 |
| SwHA931R^3^ | TCTGAAATGGGAGGCTGGTGTT |  |  |
| SwHA752F^3^ | TAGAGCCGGGAGACAAAATAACAT | HA segment 2 | 1026 |
| BmNs890R^2^ | ATATCGTCTCGTATTAGTAGAAACAAGGGTGTTTT |  |  |
| BmNA1F^2^ | TATTGGTCTCAGGGAGCAAAAGCAGGAGT | NA segment 2 | 720 |
| SwNA720R^3^ | TTACTTGGTCCATCGGTCATTACA |  |  |
| SwNA375F^3^ | CCCCTTGGAATGCAGAACCTT | NA segment 2 | 1038 |
| BmNA1413R^2^ | ATATGGTCTCGTATTAGTAGAAACAAGGAGTTTTTT |  |  |
| BmM1F^2^ | TATTCGTCTCAGGGAGCAAAAGCAGGTAG | M | 1027 |
| BmM1027R^2^ | ATATCGTCTCGTATTAGTAGAAACAAGGTAGTTTTT |  |  |
| BmNs1F^2^ | TATTCGTCTCAGGGAGCAAAAGCAGGGTG | NS | 890 |
| BmNs890R^2^ | ATATCGTCTCGTATTAGTAGAAACAAGGGTGTTTT |  |  |

^1^Protocols reported by CDC (41).

^2^Primers reported by Hoffman et al. (2021) (40).

^3^Primers kindly provided by Dr. Daniel Perez (University of Maryland, College Park, USA. Currently at College of Veterinary Medicine, University of Georgia, Athens, GA).

**Table S2.** Additional information on the Influenza A Virus (H1N1) isolates from the Virology Laboratory collection included in this study.

| **Strain Name** | **Region** | **Isolation Source** | **Collection Date** | **Global Swine H1 Clade** | **US Swine H1 Clade** | **Segment Number** | **Sequence**  **Accession** | **Influenza Segment/Protein Details IRD** |
| --- | --- | --- | --- | --- | --- | --- | --- | --- |
| A/swine/Colombia/0401/2008 | Antioquia | Nasal swabs | 10/2008 | 1A.1 | Alpha (α-H1) | 4 | JX826516 | <https://bit.ly/3lcsmXX> |
|  |  |  |  |  |  | 7 | NA | FASTA |
| A/swine/Colombia/0801/2008 | Antioquia | Nasal swabs | 10/2008 | 1A.1 | Alpha (α-H1) | 4 | NA | FASTA |
|  |  |  |  |  |  | 6 | NA | FASTA |
|  |  |  |  |  |  | 8 | MK256274 | <https://bit.ly/39njTP2> |
| A/swine/Colombia/1-01/2009 | Valle del Cauca | Nasal swabs | 08/2009 | 1A.3.3.2 | H1N1pdm09 | 4 | JX826511 | <https://bit.ly/3sySMaa> |
| A/swine/Antioquia/0201/2009 | Antioquia | Nasal swabs | 06/2009 | 1A.3.3.2 | H1N1pdm09 | 4 | NA | FASTA |
|  |  |  |  |  |  | 6 | NA | FASTA |
|  |  |  |  |  |  | 7 | MK007255 | <https://bit.ly/3PxnrPp> |
|  |  |  |  |  |  | 8 | MK007256 | <https://bit.ly/3Pji7yS> |
| A/swine/Colombia/1503/2010 | Cundinamarca | Nasal swab |  | 1A.3.3.2 | H1N1pdm09 | 4 | NA | FASTA |
|  |  |  |  |  |  | 6 | NA | FASTA |
| A/swine/ Antioquia/3-015/2015 | Antioquia | lung | 09/11/2015 | 1A.3.3.2 | H1N1pdm09 | 4 | MH997695 | <https://bit.ly/3FNo1nE> |
|  |  |  |  |  |  | 6 | MH997697 | <https://bit.ly/39OIxbk> |
|  |  |  |  |  |  | 7 | MH997696 | <https://bit.ly/3FLJ2ir> |
|  |  |  |  |  |  | 8 | MH997698 | <https://bit.ly/3PkStKc> |
| A/swine/Cundinamarca/1-019/2015 | Cundinamarca | lung | 09/18/2015 | 1A.3.3.2 | H1N1pdm09 | 4 | MH997691 | <https://bit.ly/3Pn6yqs> |
|  |  |  |  |  |  | 6 | MH997693 | <https://bit.ly/3l7Olz1> |
|  |  |  |  |  |  | 7 | MH997692 | <https://bit.ly/3wqoho3> |
|  |  |  |  |  |  | 8 | MH997694 | <https://bit.ly/39nVSr6> |
| A/swine/Antioquia /3-020/2015 | Antioquia | lung | 09/24/2015 | 1A.3.3.2 | H1N1pdm09 | 4 | MH997699 | <https://bit.ly/3Le4JJd> |
|  |  |  |  |  |  | 6 | MH997701 | <https://bit.ly/3wawNZE> |
|  |  |  |  |  |  | 7 | MH997700 | <https://bit.ly/3yDGBwY> |
|  |  |  |  |  |  | 8 | MH997702 | <https://bit.ly/39lhvbf> |
| A/swine/Antioquia /3-021/2015 | Antioquia | lung | 09/24/2015 | 1A.3.3.2 | H1N1pdm09 | 4 | MH997707 | <https://bit.ly/37HQDls> |
|  |  |  |  |  |  | 6 | MH997709 | <https://bit.ly/3PmulGQ> |
|  |  |  |  |  |  | 7 | MH997708 | <https://bit.ly/3FI4hSi> |
|  |  |  |  |  |  | 8 | MH997710 | <https://bit.ly/3yDIKZo> |
| A/swine/Antioquia /3-027/2015 | Antioquia | lung | 10/07/2015 | 1A.3.3.2 | H1N1pdm09 | 4 | MH997703 | <https://bit.ly/3FGCRfz> |
|  |  |  |  |  |  | 6 | MH997705 | <https://bit.ly/3w8UyBb> |
|  |  |  |  |  |  | 7 | MH997704 | <https://bit.ly/3laX7ws> |
|  |  |  |  |  |  | 8 | MH997706 | <https://bit.ly/3FS6Z7S> |

**Table S3.** Phylogenetic trees build parameters for HA, NA, M, and NS segments of Influenza A Virus (H1N1).

| **Segment** | **Aligned Method** | **Tree Algorithm** | **Bootstrap Analysis** | **Accessions** |
| --- | --- | --- | --- | --- |
| HA | Muscle | RaxML | 1000 replicates | DQ280219,DQ415317,CY019891,CY022125,EU116040,CY026283,CY026299,GQ161136,CY045852,CY045740,CY077942,CY080402,CY095675,CY112548,CY114429,U47305,CY012304,JX138525,CY121680,CY121448,CY116404,JX826516,JX826511,CY122349,KC703326,CY146793,CY068710,KF057117,CY157967,CY160728,CY163576,KF533050,CY177464,KJ946415,KJ889300,LN867668,KM496995,KR701376,KP335925,KX413553,KU976524,KX264750,CY207053,CY207141,KY925626,KY925038,KY925870,KU322010,MF099084,MH997691,MH997695,MH997699, MH997703,MH997707,LC431428,MK943737 |
| NA | Muscle | RaxML | 1000 replicates | CY020047,CY020295,CY021079,EU139833,CY026285,CY032215,CY044670,CY045742,FJ830857,EU502888,CY077944,CY087786,HM210862,CY085392,CY033990,AB620193,CY013815,CY112275,CY121682,CY116580,CY125190,CY116129,CY116317,CY125864,KC703323,KC703346,CY162746,CY170873,CY006341,KJ667965,CY176580,KJ946416,KM027793,KM029729,KJ889350,KR701251,GU135927,KT828232,CY207047,CY207055,CY207143,KY681622,KX571224,MF768837,MG856216,MG836813,MH997693,MH997697,LC431430,AB434402 |
| M | Muscle | RaxML | 1000 replicates | CY002177,CY026284,CY026300,CY025254,CY045741,AB573668,CY077945,CY033562,CY095678,JQ220553,JQ612486,CY112726,CY113086,M33045,CY113398,CY121262,CY125165,CY116561,CY116534,CY125863,CY130926,KC703322,KF014365,CY158314,CY157752,CY163865,CY006884,CY177465,KJ855398,KM029706,CY189768,KR701228,KR700643,KT844342,KP638025,KX004375,CY084556,CY207054,CY207118,CY207142,KX879560,KU322088,CY242092,MH061738,MF098936,MF099103,MH997692,MH997696,MH997700,MH997704,MH997708,MK007255,LC431431,MN596849,MH329000 |
| NS | Muscle | RaxML | 1000 replicates | CY016232,CY026287,CY026303,EU403422,M80965,CY038651,CY045856,CY045744,GQ422413,CY084885,CY091773,CY113065,CY112609,CY118171,CY118462,CY125200,CY116139,CY122369,CY146797,CY068413,CY157611,KF560194,CY009296,CY006703,CY173131,CY178715,KF840464,KM028707,KM028171,KJ764752,KJ943158,LC033109,KM355397,KT983799,KX004009,KX408215,KM496991,CY207057,CY207145,KP404176,KY926032,CY245973,MH061835,MF098868,MH997694,MH997698,MK007256,AB573675,LC431432,MK256274,MN596843,MH328714 |

**Tabla S4.** FASTA sequence of Influenza A Virus (H1N1) not reported previously.

| **Strain Name** | **Segment Number** | **FASTA sequence** |
| --- | --- | --- |
| A/swine/Colombia/0401/2008 | 7 | GAGCAAAAGCAGGTAGATATTTAAAGATGAGTCTTCTAaCCGAGGTCGaAACGTACGTTCTTTCTATCATCCCGTCA GGCCCCCTCAAAGCCGAGATCGCGCAGAGACTGGAAAGTGTCTTTGCAGGAAAGAACACAGATCTTGAGGCTCTCAT  GGAATGGCTAAAGACAAGACCAATCTTGTCACCTCTGACTAAGGGAATTTTAGGATTTGTGTTCACGCTCACCGTGC  CCAGTGAGCGAGGACTGCAGCGTAGACGCTTTGTCCAAAATGCCCTAAATGGGAATGGGGACCCGAACAACATGGAT  AGAGCAGTTAAACTATACAAGAAGCTCAAAAGAGAAATAACGTTCCATGGGGCCAAGGAGGTGTCACTAAGCTATTC  AACTGGTGCACTTGCCAGTTGCATGGGCCTCATATACAACAGGATGGGAACAGTGACCACAGAAGCTGCTTTTGGTC  TAGTGTGTGCCACTTGTGAACAGATTGCTGATTCACAGCATCGGTCTCACAGACAAATGGCTACTACCACCAATCCA  CTAATCAGGCATGAAAACAGAATGGTGCTGGCTAGCACTACGGCAAAGGCTATGGAACAGATGGCTGGATCGAGTGA  ACAGGCAGCAGAGGCCATGGAGGTTGCTAATCAGACTAGGCAGATGGTACATGCAATGAGAACTATTGGGACTCATC  CTAGCTCCAGTGCTGGTTGAAAGATGACCTTCTTGAAAATTTGCAGGCCTACCAGAAGCGAATGGGAGTGCAGATGC  AGCGATTCAAGTGATCCTCTCGTCATTGCAGCAAATATCATTGGGATCTTGCACCTGATATTGTGGATTACTGATCG  TCTTTTTTTCAAATGTATTTATCGTCGCTTTAAATACGGTTTGAAAAGAGGGCCTTCTACGGAAGGAGTGCCTGAGT  CCATGAGGGAAGAATATCAACAGGAACAGCAGAGTGCTGTGGATGTTGACGATGGTCATTTtGTCAACATAGAGCTA  GAGTAAAAAACTACCTTG |
| A/swine/Colombia/0801/2008 | 4 | ATGAATCCAAACCAAAAGATAATAACCATTGGTTCGATCTGTCTAATAGTTGGAATAACTAGCCTAATATTACAAAT  AGGGAATATAATCTCAATATGGATTAGCCACTCAATTCAACTTGGGAATCAAAATCAGATTGTCACATGCAACCAAA  GCATCATTACCTATGAAAACAACACATGGGTGAATCAAACATATGCTAACATTAGCAATGCTAACATTATTGCTGGA  CAGGATGCAACTTCAATGATATTAGCCGGCAATTCCTCTCTTTGCCCCATCAGTGGGTGGGCTATATACAGCAAAGA  CAATGACATAAGAATTGGTTCCAAAGGAGACATTTTTGTCATAAGAGAGCCGTTTATTTCATGCTCTCACTTGGAAT  GCAGAACCTTYTTTCTGACTCAAGGCGCTTTGCTGAATGACAAGCATTCAAATGGAACCGTCAAGGACAGAAGCCCT  TATAGAACCTTAATGAGCTGCCCTATTGGTGAAGCTCCATCTCCGTACAATTCAAGGTTCGAATCGGTTGCTTGGTC  AGCAAGTGCATGCCATGATGGCATGAGCTGGCTAACAATCGGAATTTCCGGTCCAGATAATGGAGCAGTGGCTGTAT  TAAAATACAATGGTATAATAACAGATACCATCAAAAGTTGGAGGAACAAAATATTGAGAACGCAAGAGTCTGAATGT  GCCTGTGTAAATGGTTCATGTTTTACTATAATGACCGATGGCCCAAGTAATGGGCAGGCCTCGTACAAAATCTTCAA  GATAGAGAAGGGGAGGATTATTAAATCAATTGAGTTGAATGCACCTAATTACCACTACGAGGAATGCTCCTGTTATC  CTGATGCAAGTAAAGTAATGTGTGTGTGCAGAGACAACTGGCATGGTTCGAACCGACCATGGGTGTCTTTCGATCAA  AATCTGGATTATCAAATAGGGTACATCTGCAGTGGGGTTTTCGGTGACAACCCGCGTTCCAATGATGGAACAGGCAG  CTGTGGTCCAGTGTCTTCTAATGGAGCAAATGGGGTAAAAGGATTTTCGTTTAGATATGGCAATGGTGTTTGGATAG  GAAGAACTAAAAGTATCAGTTCCAGAAACGGATTTGAGATGATTTGGGATCCTAATGGGTGGACAGAGACTGATAGT  AGTTTCTCTGTGAAACAAGATATTGTAGCAATAACTGATTGGTCAGGGTACAGCGGGAGTTTCGTTCAACATCCTGA  ACTAACAGGACTGGACTGCATAAGGCCTTGCTTCTGGGTTGAGTTAATCAGAGGACAACCTAAGGAGAACACAATCT  GGACTAGTGGGAGCAGCATTTCCTTTTGTGGCGTGAATAGTGATACTGTAGGCTGGTCTTGGCCAGACGGCGCTGAG  TTGCCATTCACCATTGACAAGTAG |
|  | 6 | ATGAATCCAAACCAAAAGATAATAACCATTGGTTCGATCTGTCTAATAGTTGGAATAACTAGCCTAATATTACAAATAGG  GAATATAATCTCAATATGGATTAGCCACTCAATTCAACTTGGGAATCAAAATCAGATTGTCACATGCAACCAAAGCATCA  TTACCTATGAAAACAACACATGGGTGAATCAAACATATGCTAACATTAGCAATGCTAACATTATTGCTGGACAGGATGCA  ACTTCAATGATATTAGCCGGCAATTCCTCTCTTTGCCCCATCAGTGGGTGGGCTATATACAGCAAAGACAATGACATAAG  AATTGGTTCCAAAGGAGACATTTTTGTCATAAGAGAGCCGTTTATTTCATGCTCTCACTTGGAATGCAGAACCTTYTTTC  TGACTCAAGGCGCTTTGCTGAATGACAAGCATTCAAATGGAACCGTCAAGGACAGAAGCCCTTATAGAACCTTAATGAGC  TGCCCTATTGGTGAAGCTCCATCTCCGTACAATTCAAGGTTCGAATCGGTTGCTTGGTCAGCAAGTGCATGCCATGATGG  CATGAGCTGGCTAACAATCGGAATTTCCGGTCCAGATAATGGAGCAGTGGCTGTATTAAAATACAATGGTATAATAACAG  ATACCATCAAAAGTTGGAGGAACAAAATATTGAGAACGCAAGAGTCTGAATGTGCCTGTGTAAATGGTTCATGTTTTACT  ATAATGACCGATGGCCCAAGTAATGGGCAGGCCTCGTACAAAATCTTCAAGATAGAGAAGGGGAGGATTATTAAATCAAT  TGAGTTGAATGCACCTAATTACCACTACGAGGAATGCTCCTGTTATCCTGATGCAAGTAAAGTAATGTGTGTGTGCAGAG  ACAACTGGCATGGTTCGAACCGACCATGGGTGTCTTTCGATCAAAATCTGGATTATCAAATAGGGTACATCTGCAGTGGG  GTTTTCGGTGACAACCCGCGTTCCAATGATGGAACAGGCAGCTGTGGTCCAGTGTCTTCTAATGGAGCAAATGGGGTAAA  AGGATTTTCGTTTAGATATGGCAATGGTGTTTGGATAGGAAGAACTAAAAGTATCAGTTCCAGAAACGGATTTGAGATGA  TTTGGGATCCTAATGGGTGGACAGAGACTGATAGTAGTTTCTCTGTGAAACAAGATATTGTAGCAATAACTGATTGGTCA  GGGTACAGCGGGAGTTTCGTTCAACATCCTGAACTAACAGGACTGGACTGCATAAGGCCTTGCTTCTGGGTTGAGTTAAT  CAGAGGACAACCTAAGGAGAACACAATCTGGACTAGTGGGAGCAGCATTTCCTTTTGTGGCGTGAATAGTGATACTGTAG  GCTGGTCTTGGCCAGACGGCGCTGAGTTGCCATTCACCATTGACAAGTAG |
| A/swine/Antioquia/0201/2009 | 4 | AACAAAAGCAGGGGAAAACAAAAGCAACAAAAATGAAGGCAATACTAGTAGTTCTGCTATATACATTTGCAACCACAAA  TGCAGACACATTATGTATAGGTTATCATGCGAACAATTCAACAGACACTGTAGACACAGTACTAGAAAAGAATGTAACA  GTAACACACTCTGTTAACCTTCTAGAAGACAAGCATAACGGGAAACTATGCAAACTAAGAGGGGTAGCCCCATTGCATT  TGGGTAAATGTAACATTGCTGGCTGGATCCTGGGAAATCCAGAGTGTGAATCACTCTCCACAGCAAGCTCATGGTCCTA  CATTGTGGAAACATCTAGTTCAGACAATGGAACGTGTTACCCAGGAGATTTCATCGATTATGAGGAGCTAAGAGAGCAA  TTGAGCTCAGTGTCATCATTTGAAAGGTTTGAGATATTCCCCAAGACAAGTTCATGGCCCAATCATGACTCGAACAAAG  GTGTAACGGCAGCATGTCCTCATGCTGGAGCAAAAAGCTTCTACAAAAATTTAATATGGCTAGTTAAAAAAGGAAATTC  ATACCCAAAGCTCAGCAAATCCTACATTAATGATAAAGGGAAAGAAATCCTCGTGCTATGGGGCATTCACCATCCATCT  ACTAGTGCTGACCAACAAAGTCTCTATCAGAATGCAGATGCATATGTTTTTGTGGGGACATCAAGATACAGCAAGAAGT  TCAAGCCGGAAATAGCAATAAGACCCAAAGTGAGGGATCGAGAAGGGAGAATGAACTATTACTGGACACTAGTAGAGCC  GGGAGACAAAATAACATTCGAAGCAACTGGAAATCTAGTGGTACCGAGATATGCATTCGCAATGGAAAGAAATGCTGGA  TCTGGTATTATCATTTCAGATACACCAGTCCACGATTGCAATACAACTTGTCAGACACCCAAGGGTGCTATAAACACCA  GCCTCCCATTTCAGAATATACATCCGATCACAATTGGAAAATGTCCAAAATATGTAAAAAGCACAAAATTGAGACTGGC  CACAGGATTGAGGAATGTCCCGTCTATTCAATCTAGAGGCCTATTTGGGGCCATTGCCGGTTTCATTGAAGGGGGATGG  ACAGGGATGGTAGATGGATGGTACGGTTATCACCATCAAAATGAGCAGGGGTCAGGATATGCAGCCGACCTGAAGAGCA  CACAGAATGCCATTGACGAGATTACTAACAAAGTAAATTCTGTTATTGAAAAGATGAATACACAGTTCACAGCAGTAGG  TAAAGAGTTCAACCACCTGGAAAAAAGAATAGAGAATTTAAATAAAAAAGTTGATGATGGTTTTCTGGACATTTGGACT  TACAATGCCGAACTGTTGGTTCTATTGGAAAATGAAAGAACTTTGGACTACCACGATTCAAATGTGAAGAACTTATATG  AAAAGGTAAGAAGCCAGTTAAAAAACAATGCCAAGGAAATTGGAAACGGCTGCTTTGAATTTTACCACAAATGCGATAA  CACGTGCATGGAAAGTGTCAAAAATGGGACTTATGACTACCCAAAATACTCAGAGGAAGCAAAATTAAACAGAGAAGAA  ATAGATGGGGTAAAGCTGGAATCAACAAGGATTTACCAGATTTTGGCGATCTATTCAACTGTCGCCAGTTCATTGGTAC  TGGTAGTCTCCCTGGGGGCAATCAGTTTCTGGATGTGCTCTAATGGGTCTCTACAGTGTAGAATATGTATTTAACATTA  GGATTTCAGAAGCATGAGAAAAACACCCTTGTTTCTACTAATACGAGACGATATA |
|  | 6 | TCAGGGAGCAAAAGCAGGAGTTTAAAATGAATCCAAACCAAAAGATAATAACCATTGGTTCGGTCTGTATGACAATTGG  AATGGCTAACTTAATATTACAAATTGGAAACATAATCTCAATATGGATTAGCCACTCAATTCAACTTGGGAATCAAAAT  CAGATTGAAACATGCAATCAAAGCGTCATTACTTATGAAAACAACACTTGGGTAAATCAGACATATGTTAACATCAGCA  ACACCAACTTTGCTGCTGGACAGTCAGTGGTTTCCGTGAAATTAGCGGGCAATTCCTCTCTCTGCCCTGTTAGTGGATG  GGCTATATACAGTAAAGACAACAGTATAAGAATCGGTTCCAAGGGGGATGTGTTTGTCATAAGGGAACCATTCATATCA  TGCTCCCCCTTGGAATGCAGAACCTTCTTCTTGACTCAAGGGGCCTTGCTAAATGACAAACATTCCAATGGAACCATTA  AAGACAGGAGCCCATATCGAACCCTAATGAGCTGTCCTATTGGTGAAGTTCCCTCTCCATACAACTCAAGATTTGAGTC  AGTCGCTTGGTCAGCAAGTGCTTGTCATGATGGCATCAATTGGCTAACAATTGGAATTTCTGGCCCAGACAATGGGGCA  GTGGCTGTGTTAAAGTACAACGGCATAATAACAGACACTATCAAGAGTTGGAGAAACAATATATTGAGAACACAAGAGT  CTGAATGTGCATGTGTAAATGGTTCTTGCTTTACTGTAATGACCGATGGACCAAGTGATGGACAGGCCTCATACAAGAT  CTTCAGAATAGAAAAGGGAAAGATAGTCAAATCAGTCGAAATGAATGCCCCTAATTATCACTATGAGGAATGCTCCTGT  TATCCTGATTCTAGTGAAATCACATGTGTGTGCAGGGATAACTGGCATGGCTCGAATCGACCGTGGGTGTCTTTCAACC  AGAATCTGGAATATCAGATAGGATACATATGCAGTGGGATTTTCGGAGACAATCCACGCCCTAATGATAAGACAGGCAG  TTGTGGTCCAGTATCGTCTAATGGAGCAAATGGAGTAAAAGGATTTTCATTCAAATACGGCAATGGTGTTTGGATAGGG  AGAACTAAAAGCATTAGTTCAAGAAACGGTTTTGAGATGATTTGGGATCCGAACGGATGGACTGGGACAGACAATAACT  TCTCAATAAAGCAAGATATCGTAGGAATAAATGAGTGGTCAGGATATAGCGGGAGTTTTGTTCAGCATCCAGAACTAAC  AGGGCTGGATTGTATAAGACCTTGCTTCTGGGTTGAACTAATCAGAGGGCGACCCAAAGAGAACACAATCTGGACTAGC  GGGAGCAGCATATCCTTTTGTGGTGTAAACAGTGACACTGTGGGTTGGTCTTGGCCAGACGGTGCTGAGTTGCCATTTA  CCATTGACAAGTAATTTGTTCAAAAAACTCCT |
| A/swine/Colombia/1503/2010 | 4 | ATGAAGGCAATACTAGTAGTTCTGCTATATACATTTGCAACCGCAAATGCAGACACATTATGTATAGGTTATCATGCGAA  CAATTCAACAGACACTGTAGACACAGTACTAGAAAAGAATGTAACAGTAACACACTCTGTTAACCTTCTAGAAGACAAGC  ATAACGGGAAACTATGCAAACTAAGAGGGGTAGCCCCATTGCATTTGGGAAAATGTAACATTGCTGGCTGGATCCTGGGA  AATCCAGAGTGTGAATCACTCTCCACAGCAAGCTCATGGTCCTACATGGTGGAATCATCTAGTTCAGACAATGGAACGTG  TTACCCAGGAGATTTCATCGATTATGAGGAGCTAAGAGAGCAATTGAGCTCAGTGTCATCATTTGAAAAGTTTGAGATAT  TCCCCAAGACAAGTTCATGGCCTAATCATGACTCGAACAAAGGTGTAACGGCAGCATGTCCTCATGCTGGAGCAAGAAGC  TTCTACAAAAATTTAATATGGCTAGTTAAAAAAGGAAATTCATACCCAAAGCTCAGCAAATCCTACATTAATGATAAAGG  GAAAGAAGTCCTCGTGCTATGGGGCATTCACCATCCATCTACTAGTGCTGACCAACAAAGTCTCTATCAGAATGCAGATG  CATATGTTTTTGTGGGGACATCAAGATACAGCAAGAAGTTCAAGCCAGAAATAGCAATAAGACCCAAAGTGAGGGATCAA  GAAGGGAGAATGAACTATTACTGGACACTAGTAGAACCGGGAGACAAAATAACATTCGAAGCAACTGGAAATCTAGTGGT  ACCGAGATATGCATTCGCAATGGAAAGAAATGCTGGATCTGGTATTATCATTTCAGATACACCAGTCCACGATTGCAATA  CAACTTGTCAGACACCCAAGGGTGCTATAAACACCAGCCTACCATTTCAGAATATACATCCGATCACAATTGGAAAATGT  CCAAAATATGTAAAAAGCACAAAATTGAGACTGGCCACAGGATTGAGGAATGTCCCGTCTATTCAATCTAGAGGCCTATT  TGGGGCCATTGCCGGTTTCATTGAAGGGGGATGGACAGGGATGGTAGATGGATGGTACGGTTATCACCATCAAAATGAGC  AGGGGTCAGGATATGCAGCCGACCTGAAGAGCACACAGAATGCCATTGACGAGATTACTAACAAAGTAAATTCTGTTATT  GAAAAGATGAATACACAGTTCACAGCAGTAGGTAAAGAGTTCAACCACCTGGAAAAAAGAATAGAGAATTTAAATAAAAA  AGTTGATGATGGTTTCCTGGACATTTGGACTTACAATGCCGAACTGTTGGTTCTATTGGAAAATGAAAGAACTTTGGACT  ACCACGATTCAAATGTGAAGAACTTATATGAAAAGGTAAGAAGCCAGTTAAAAAACAATGCCAAGGAAATTGGAAACGGC  TGCTTTGAATTTTACCACAAATGCGATAACACGTGCATGGAAAGTGTCAAAAATGGGACTTATGAATACCCAAAATACTC  AGAGGAAGCAAAATTAAACAGAGAAGAAATAGATGGGGTAAAGCTGGAATCAACAAGGATTTACCAGATTTTGGCGATCT  ATTCAACTGTCGCCAGTTCATTGGTACTGGTAGTCTCCCTGGGGGCAATCAGTTTCTGGATGTGTTCTAATGGGTCTCTA  CAGTGTAGAATATGTATTTAA |
|  | 6 | ATGAATCCAAACCAAAAGATAATAACCATTGGTTCGGTCTGTATGACAATTGGAATGGCTAACTTAATATTACAAAT  TGGAAACATAATCTCAATATGGATTAGCCACTCAATTCAACTTGGGAATCAAAATCAGATTGAAACATGCAATCAAA  GCGTCATTACTTATGAAAACAACACTTGGGTAAATCAGACATATGTTAACATCAGCAACACCAACTTTGCTGCTGGA  CAGTCAGTGGTTTCCGTGAAAATAGCGGGCAATTCCTCTCTCTGCCCTGTTAATGGATGGGCTATATACAGTAAAGA  CAACAGTATAAGAATCGGTTCCAAGGGGGATGTGTTTGTCATAAGGGAACCATTCATATCATGCTCCCCCTTGGAGT  GCAGAACCTTCTTCTTGACTCAAGGGGCCTTACTAAATGACAAACATTCCAATGGAACCATTAAAGACAGGAGCCCA  TATCGAACCCTAATGAGCTGTCCTATTGGTGAAGTTCCCTCTCCATACAACTCAAGATTTGAGTCAGTCGCTTGGTC  AGCAAGTGCTTGTCATGATGGCATCAATTGGCTAACAATTGGAATTTCTGGCCCAGACAATGGGGCAGTGGCTGTGT  TAAAGTACAACGGCATAATAACAGACACTATCAAGAGTTGGAGAAACAATATATTGAGAACACAAGAGTCTGAATGT  GCATGTGTAAATGGTTCTTGCTTTACTGTAATGACCGATGGACCAAGTGATGGACAGGCCTCATACAAGATCTTCAG  AATAGAAAAGGGAAAGATAGTTAAATCAGTCGAAATGAATGCCCCTAATTATCACTATGAGGAATGCTCCTGTTATC  CTGATTCTAGTGAAATCACATGTGTGTGCAGGGATAACTGGCATGGCTCGAATCGACCGTGGGTGTCTTTCAACCAG  AATCTGGAATATCAGATAGGATACATATGCAGTGGGATTTTCGGAGACAATCCACGCCCCAATGATAAGACAGGCAG  TTGTGGTCCAGTATCGTCTAATGGAGCAAATGGAGTAAAAGGATTTTCATTCAAATACGGTAATGGTGTTTGGATAG  GGAGAACTAAAAGCATTAGTTCAAGAAGCGGTTTTGAGATGATTTGGGATCCGAACGGATGGACTGGGACAGACAAT  AACTTCTCAATAAAGCAAGATATCGTAGGAATAAATGAGTGGTCAGGATATAGCGGGAGTTTTGTTCAGCATCCAGA  ACTAACAGGGCTGGATTGTATAAGACCTTGCTTCTGGGTTGAACTAATCAGAGGGCGACCCAAAGAGAACACAATCT  GGACTAGCGGGAGCAGCATATCCTTTTGTGGTGTAAACAGTGACACTGTGGGTTGGTCTTGGCCAGACGGTGCTGAG  TTGCCATTTACCATTGACAAGTAA |
